# Combining a Modified Particle Filter Method and Indoor Magnetic Fingerprint Map to Assist Pedestrian Dead Reckoning for Indoor Positioning and Navigation

**DOI:** 10.3390/s20010185

**Published:** 2019-12-28

**Authors:** Fang-Shii Ning, Yu-Chun Chen

**Affiliations:** Department of Land Economics, National Chengchi University, Taipei 11605, Taiwan; 106257031@nccu.edu.tw

**Keywords:** indoor positioning, magnetic fingerprint map, pedestrian dead reckoning, particle filter

## Abstract

Although advancement has been observed in global navigation satellite systems and these systems are widely used, they cannot provide effective navigation and positioning services in covered areas and areas that lack strong signals, such as indoor environments. Therefore, in recent years, indoor positioning technology has become the focus of research and development. The magnetic field of the Earth is quite stable in an open environment. Due to differences in building and internal structures, this type of three-dimensional vector magnetic field is widely available indoors for indoor positioning. A smartphone magnetometer was used in this study to collect magnetic field data for constructing indoor magnetic field maps. Moreover, an acceleration sensor and a gyroscope were used to identify the position of a mobile phone and detect the number of steps travelled by users with the phone. This study designed a procedure for measuring the step length of users. All obtained information was input into a pedestrian dead reckoning (PDR) algorithm for calculating the position of the device. The indoor positioning accuracy of the PDR algorithm was optimised using magnetic gradients of magnetic field maps with a modified particle filter algorithm. Experimental results reveal that the indoor positioning accuracy was between 0.6 and 0.8 m for a testing area that was 85 m long and 33 m wide. This study effectively improved the indoor positioning accuracy and efficiency by using the particle filter method in combination with the PDR algorithm with the magnetic fingerprint map.

## 1. Introduction

Outdoor positioning performance has approached perfection due to the global navigation satellite system (GNSS). Moreover, in the past decade, research and development was focused on indoor positioning [1]. According to the 2015 US survey, approximately 70%–80% of the population use mobile personal navigation services through smartphones [2]. Thus, there is a potential for developing indoor positioning and navigation conducted using a mobile phone.

GNSS signals are affected by the shadowing of building indoors. Moreover, Wi-Fi, iBeacon, and RFID require additional infrastructure and regular maintenance, which is expensive. The inertial navigation system (INS) and pedestrian dead reckoning (PDR) algorithms do not require the collection of external signals. However, errors accumulate rapidly over time when these algorithms are used. Moreover, the accuracy of image positioning is high, but the image processing method is complex and requires high-performance hardware. Magnetic positioning does not require additional hardware equipment, but its accuracy is lower than that of image positioning. Each of the aforementioned technology has its own advantages and disadvantages. Currently, there is no single technology that provides all necessary factors. We should identify user requirements and combine different technologies to address those requirements with the lowest cost, highest range, and highest accuracy possible. Table 1 presents the current indoor positioning technologies [1], and Table 2 compares the more frequent use of wireless indoor position technologies [3].

The Microsoft Indoor Localization Competition of 2016 reported the accuracy of fusion of geomagnetism and Wi-Fi was better than many other positioning methods [4]. This is meaning geomagnetic signal map is expected to exceed the traditional Wi-Fi to distinguish fingerprints in different locations [5]. The use of magnetic field measurements, unlike typical Wi-Fi or Bluetooth positioning measurements, are unaffected by moving humans, providing more time-invariant location information [5]. Based on the aforementioned factors, a smartphone magnetometer was used in this study to collect magnetic field data for constructing indoor magnetic fingerprint maps, and an acceleration sensor and a gyroscope were used to record the position of a mobile phone and detect the number of steps taken by a user with the smartphone. All obtained information was input into the PDR algorithm to calculate the position of the smartphone [6,7,8]. Compared with other wireless positioning systems, PDR has an easier operation and a lower cost for common users because no additional equipment is required. However, PDR results estimated from inertial measurement unit (IMU) data have errors that are accumulated over time. Thus, many methods have been proposed for correcting PDR positioning errors, such as combining various sensors and wireless devices for error correction [2,3] and conducting algorithmic advancements for obtaining heading direction and step length estimations [9,10,11,12,13,14,15,16]. This study optimised the PDR algorithm by using the difference magnetic fingerprint between real-time measurement and magnetic fingerprint map data to calculate the weight then put in a particle filter method (in this study call modified particle filter) to get the position of user. It does not need extra devices just using a smartphone to achieve indoor navigation.

## 2. Methodology

**Figure 1:**
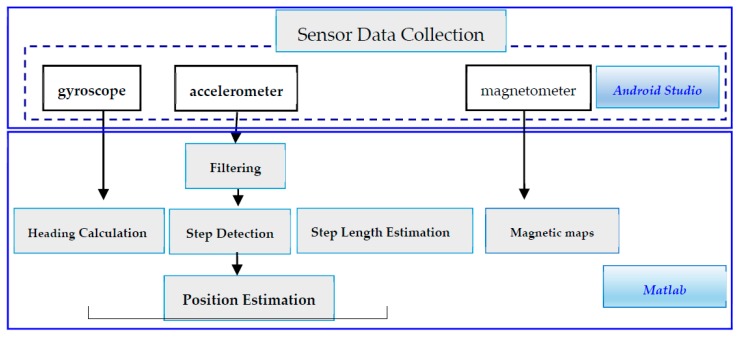
Sensors used in the structure involving the PDR algorithm and magnetic maps.

Three types of sensors were used in this study—a gyroscope, an accelerometer, and a magnetometer. Data from the three sensors were used for calculating heading direction, conducting step detection, and creating magnetic maps, respectively (Figure 1).

Android Studio was used for developing the test environment and for transferring data from sensors. Sensor data were transferred to a computer through a mobile entity. Then, MATLAB (Mathworks, Natick, MA, USA) was used for compiling the algorithm and illustrating results. In other words, sensor data collection and PDR algorithm compilation were performed using Android Studio and MATLAB, respectively.

### 2.1. Accelerometer Data and Pace Detection

When a smartphone is placed on a flat surface, accelerometer data contain intense vibrations, which represent noise. Therefore, accelerometer data have to be smoothed using a filter. Ning compared the accuracies of the Savitzky–Golay (SG) filter and fast Fourier transform (FFT) for smoothing pace detection data [16]. The result revealed that the SG filter is more accurate than FFT. Moreover, as the pace increased, the rate could be reduced to 1.1% [16]. The SG filter was proposed in 1964 [17] and has been widely used for smoothing data and filtering noise. The advantage of this filter is that the shape of data and the width of each wave are retained while filtering noises. Thus, during the data smoothing process, the number of waves of smoothed data is equal to that of raw data.

Consequently, Equation (1) is used in this study for computing the resultant acceleration force of the three axes ($Acc_{value}$), and the SG filter was selected for smoothing data:

(1) $$Acc_{value}=\;\sqrt{a_{x}{}^{2}+a_{y}{}^{2}+a_{z}{}^{2}}$$

**Figure 2:**
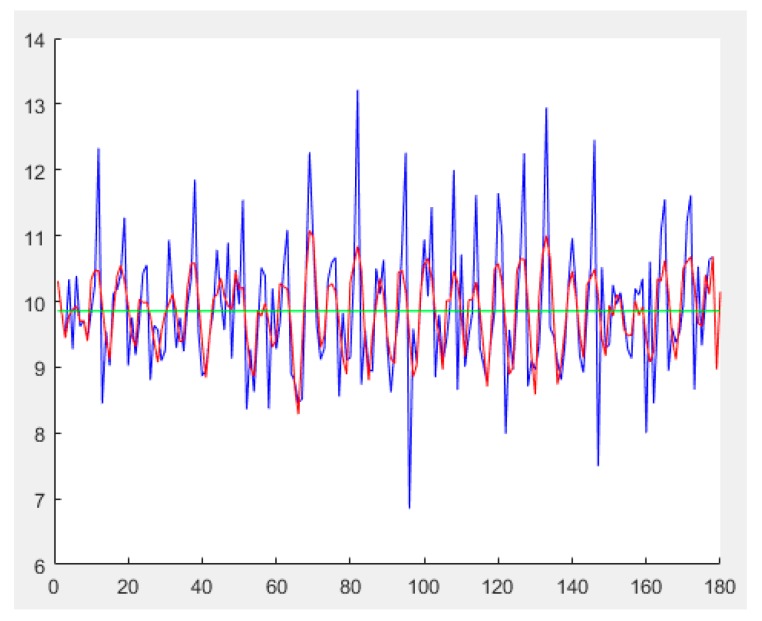
Signals before and after smoothing using the SG filter and the threshold (blue: raw data, red: after smoothing, green: threshold).

In Figure 2, the vertical axis presents acceleration values, the horizontal axis presents the time axis, and the red line denotes the result of smoothed data. Although the shapes of smoothed data do not vary to a great extent, some incorrect peaks have to be filtered using time setting and the wave crest threshold (green line). The wave crest threshold is the average of the total acceleration, 9.8528 m/s^2^. Moreover, the points at which every complete waves and the threshold value firstly intersect are viewed as the timing when feet are overlapped during walking and as the time points of estimating directions.

### 2.2. Heading Direction Calculation

**Figure 3:**
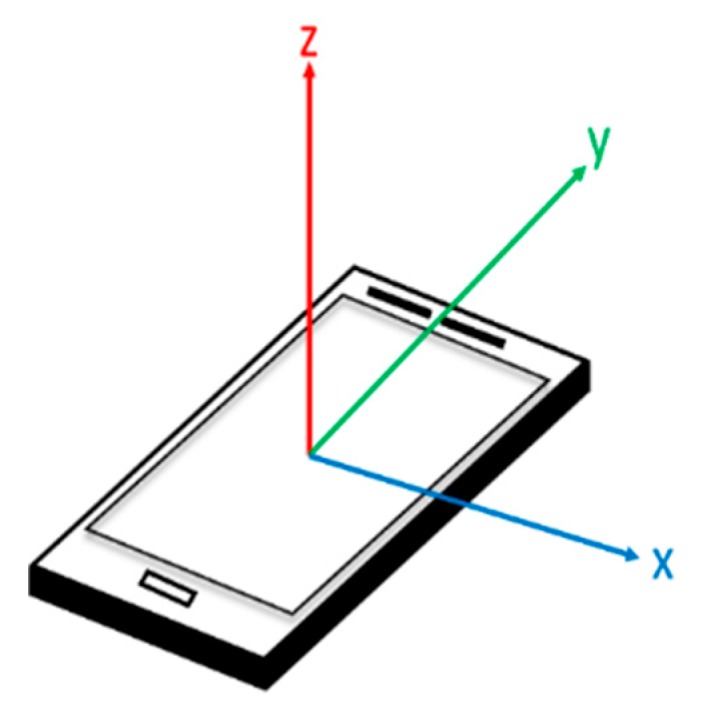
Three axes of the smartphone.

**Figure 4:**
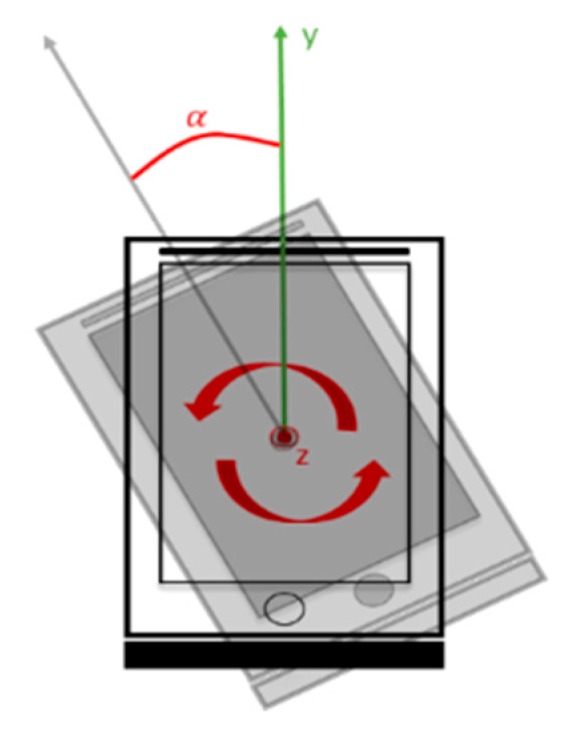
Angle of rotation of the *z*-axis of the mobile device.

Heading direction calculation is very important then high-density data are required to compute rotation angles at every moment. Data are obtained in terms of the angular velocity of the axis (as shown in Figure 3 and Figure 4). The angle was the first-order integral of the angular velocity, and the concurrent angle changes were identified through the integration of the sampling time and angular velocity data (Equation (2)) [18]. Therefore, the obtained data have to be converted into the corresponding angle format while calculating the rotation of the *z* axis for calculating follow-up coordinates.

(2) $$Angular\;Velocity\;\left(\frac{radian}{s}\right)=\frac{Angle}{Time}$$

### 2.3. Step Length

The estimation of the step length is often based on the length of the leg and the walking frequency [19]. However, considering the reality, the user does not know his or her leg length or walking frequency. Therefore, this study designs a function “Step Count” key in the program after walking a known distance, the program will record the current time and acceleration value, in the follow-up processing to determine the number of steps that users walk during this period, using Equation (3) to initially calculate the user’s step length, as the basis for subsequent positioning calculation:

(3) $$Step\;Length=\;\frac{Distance}{Step}$$

### 2.4. Pedestrian Dead Reckoning

**Figure 5:**
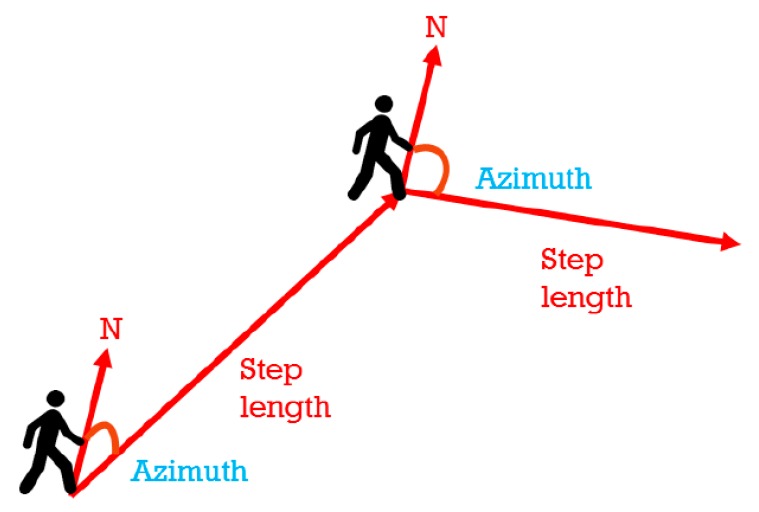
Concept of PDR.

PDR is a relative positioning technology that estimates the positions of users through moving distances and direction observations that are conducted from the initial positions of users by using inertial sensors (Figure 5).

PDR can be expressed as follows:

$$X_{t}=\;X_{t-1}+{\hat{s}}_{\left[t-1,t\right]}\sin\Psi_{\left[t-1,\;t\right]}$$

(4) $$Y_{t}=\;Y_{t-1}+{\hat{s}}_{\left[t-1,t\right]}\cos\Psi_{\left[t-1,\;t\right]}\;$$

where $\left(X_{t},\;Y_{t}\right)\;\mathrm{and}\;\left(X_{t-1},\;Y_{t-1}\right)$ represent coordinates at time *t* and (*t* − 1), respectively. Here, ${\hat{s}}_{\left[t-1,t\right]}$ represents the moving distance from (*t* − 1) to *t*, which was defined as the length of a user’s step in this study. Moreover, $\Psi_{\left[t-1,\;t\right]}$ indicates a user’s moving direction at (*t* − 1) which was defined as user’s heading direction in this study.

### 2.5. Magnetic Field Intensity Values and Fingerprint Recognition Elements 

**Figure 6:**
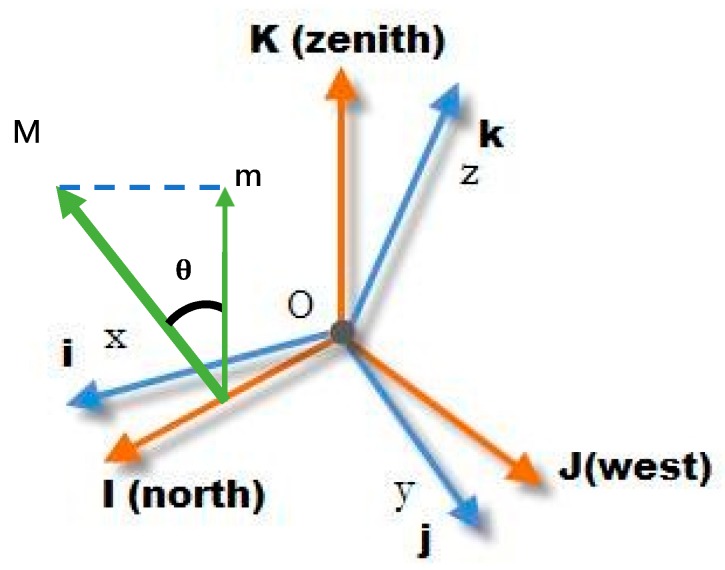
Vertical and horizontal components obtained from gravity.

A magnetic signal has an obvious disadvantage that less number of fingerprint recognition elements are available for use. When the relationship between the coordinate system of the acceleration sensor and international terrestrial reference system (ITRS) is uncertain, the directions of gravity can only be detected using an acceleration sensor. However, the number of the fingerprint recognition elements of the sensor decrease to two—gravitational direction and horizontal direction. In Figure 6, blue arrows represent the coordinates of a mobile sensor, the orange arrows represent the coordinates of the ITRS, *M* denotes the magnetic direction, and *m* represents the magnetic component of the reverse direction of acceleration.

Though the relationships between sensor coordinates system, ITRS, and the true north are unknown, the components of XY plane are the same, despite the true north. This study employs the INS signal and magnetic field intensity values to calculate the angle (θ) between the directions of the magnetic field and gravity through Equation (5) and to determine magnetic components in the direction of gravity and the horizon [20]:

(5) $$\cos\left(\theta \right)=\;\frac{m_{x}M_{x}+\;m_{y}M_{y}+\;m_{z}M_{z}}{\sqrt{m_{x}^{2}+m_{y}^{2}+m_{z}^{2}}\sqrt{M_{x}^{2}+M_{y}^{2}+M_{z}^{2}}}$$

### 2.6. Magnetic Field Positioning

The inspiration for magnetic positioning was attained from the fact that animals rely on the magnetic field of the Earth to locate their destination. Moreover, an indoor environment has steel structures that provide a unique spatially varying environmental magnetic field that is used for positioning. Animals use the magnetic field of the Earth in a similar manner, but the spatial scale is smaller [21]. In positioning and navigation applications, the magnetic field is used to determine the azimuth angle or heading direction [22]. However, a magnetic field anomaly exists in indoor environments. Thus, determining the accurate heading direction is difficult. Fingerprint recognition methods can use these magnetic field anomalies constructively [20]. In fact, the more obvious the abnormality is, the more unique is the magnetic “fingerprint,” the more features are attained in the fingerprint, and the better is the positioning result. Gozick et al. collected 2000 data samples by using mobile phones with built-in magnetometers, identified some landmarks, and created a magnetic map of multiple floors in a building [22].

### 2.7. Particle Filter

Issues, such as the possibility of having similar magnetic values in places that are far apart, how to determine the current location of a user, and how to set the search range of fingerprint identification, should be discussed. Le Grand and Thrun directly used information collected using a mobile device for obtaining the particle state at each time breakpoint (*t*) and for determining the current state of a user by assigning appropriate weights to parameters, such as velocity *v*, angular velocity ω, location (*x*, *y*), and direction *θ* (Equation (6)) [23]:

(6) $$\left\{\begin{array}{c} v(t+\Delta t)=v(t)+\varepsilon_{acceleration}^{linear}\Delta t\\ \omega (t+\Delta t)=\omega (t)+\;\varepsilon_{acceleration}^{angular}\Delta t\\ \left[\begin{array}{c} x(t+\Delta t)\\ y(t+\Delta t) \end{array}\right]=\left[\begin{array}{c} x(t)\\ y(t) \end{array}\right]+\left[\begin{array}{c} \cos(\theta (t))\\ \sin(\theta (t)) \end{array}\right]v(t+\Delta t)\Delta t\\ \theta (t+\Delta t)=\theta (t)+\omega (t+\Delta t)\Delta t \end{array}\right\}$$

Xie et al. (2015) used a mobile device to collect information for determining the particle state. In their study, the velocity *v* and angular velocity ω data were eliminated, and instead, step *l* data were used as the basis for calculating the next coordinate by using Equation (7) to cause the particle to enter the next time breakpoint state. The step length that best conforms to the user’s current state can be determined by adding the Gaussian distribution noise ($G_{\theta }\;and\;G_{l}$), increasing the particle search range, assigning appropriate weight through collected magnetic values, and by finally weighting the step state to its expectations [24].

(7) $$\{\begin{array}{c} \theta_{i}^{t+1}=\;\theta_{i}^{t}+\;∆\theta +\;G_{\theta }\\ \left[\begin{array}{c} x_{i}^{t+1}\\ y_{i}^{t+1} \end{array}\right]=\;\left[\begin{array}{c} x_{i}^{t}\\ y_{i}^{t} \end{array}\right]+\;\left[\begin{array}{c} \cos\left(\theta_{i}^{t+1}\right)\\ \sin\left(\theta_{i}^{t+1}\right) \end{array}\right]*\left(l\;+\;G_{l}\right) \end{array}$$

In the study conducted by Xie et al. (2015), weight is computed by comparing the magnetic value of each particle obtained through observation and the value in the magnetic field database for calculating the covariance matrix. Finally, Equation (8) is used to assign a different weight to each particle, where *w* is the weight, *z* is the observation measurement, *s* is a certain state in the magnetic field database, and *n* is the order of *z*. Here, *V* represents the covariance matrix obtained after comparing observed magnetic value and the value in the database for each particle [24]:

(8) $$\begin{array}{l} w_{i}^{t+1}=P(z^{t+1}-z^{t}|s_{i}^{t},s_{i}^{t=1})\\ =\frac{1}{{\left(2\pi \right)}^{\frac{n}{2}}{\left|V\right|}^{\frac{1}{2}}}\exp\left\{-\frac{1}{2}{\left[\left(z^{t+1}-z^{t})-(obv(s_{i}^{t+1})-obv(s_{i}^{t}\right)\right]}^{T}V^{-1}\left[\left(z^{t+1}-z^{t})-(obv(s_{i}^{t+1})-obv(s_{i}^{t}\right)\right]\right\} \end{array}$$

The particle filter method was used to avoid any difference between values obtained during the establishment phase of the fingerprint map database and positioning stages. Moreover, fingerprint differences were used to assign appropriate weights to each particle for obtaining coordinates and orientation that best match the user’s state. Information required at the beginning of the positioning process includes the initial position, orientation, and step length. The step length estimation is often based on users’ leg lengths and walking frequencies. However, in a practical scenario, users usually do not know their leg length or walking frequency. Thus, the step length of users can be estimated by calculating the number of steps taken between two points. In this study, a function was designed in the programme to allow a user to walk a particular distance. Then, in follow-up processing steps, the number of steps in the route and the user’s step length were calculated. Weight is a crucial parameter in the particle filter method. Equation (8) is proposed by Xie et al. 2015 [24], when it used in this study’s testing area encountering a significant turn will not be able to carry out. After examination found that is the weighting problem. It will overweight the position near the wall then cannot make the turn. Therefore, this study modified the weighting model and named the modified particle filter. The modified particle filter weighting model is shown in Equation (9):

(9) $$\begin{array}{l} w_{i}^{t+1}=P(z^{t+1}-z^{t}|s_{i}^{t},s_{i}^{t=1})\\ =\left|\frac{1}{{(z}^{t+1}-z^{t})-(\mathrm{obv}(s_{i}^{t+1})-\mathrm{obv}(s_{i}^{t}))}\right| \end{array}$$

In the weight distribution conducted using Equation (9), $z^{t}\;\mathrm{and}\;z^{t+1}$ represent the magnetic fingerprint values at *t* and *t* + 1 collected at testing area, respectively, and $obv\left(s_{i}^{t}\right)\;\mathrm{and}\;obv\left(s_{i}^{t+1}\right)$ denote the *t* and *t* + 1 coordinates of magnetic fingerprint values in the magnetic field fingerprint map database. This weighting model is the absolute value of the inverse value of the difference between the actual magnetic fingerprint observed *t* and *t +* 1 time positions and the difference in the magnetic fingerprint database.

During the particle filter calculation, each step state may be updated due to the orientation and step accumulation error or the relationship with the wall; thus, some particles will be estimated to be in the magnetic fingerprint map database where there is no data, such as those of a wall, room, or elevator. Therefore, a resampling mechanism should be incorporated in the algorithm. Particles that are not in the walking range of a user or appear at an impossible location should be removed, and remaining particles should be resampled. Resampled particles should not be excessively concentrated at a certain position. Therefore, sampling should be conducted at the weighted average position of remaining particles by using the normal distribution method so that the estimated trajectory can be reversed to the normal trajectory. Finally, all particles are calculated using the weighted average method (Equation (10)), including *x* coordinates, *y* coordinates, azimuth angle, and step size [20]:

(10) $$\hat{s}=\;\overset{N}{\underset{i=1}{\sum }}s_{i}\cdot w_{i}$$

## 3. Study Area and Data Collection 

This study used a combination of the particle filter method and magnetic field maps to assist PDR for indoor positioning. Thus, it is necessary to collect indoor magnetic field fingerprint data for creating an environmental magnetic field fingerprint map database. Then, the modified particle filter method that is proposed in this study was used. In this method, the magnetic field fingerprint database and PDR were used together for indoor navigation. Results collected in the experimental area and magnetic field fingerprint database are introduced in this section.

### 3.1. Experimental Area

**Figure 7:**
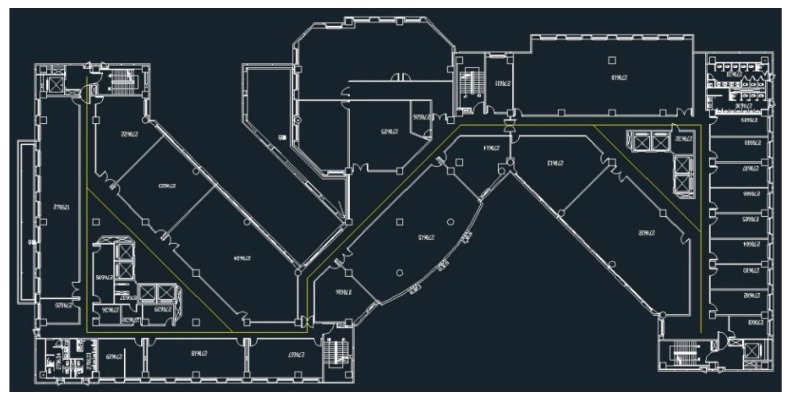
Floor plan of the sixth floor of the general building.

The experimental area of this study was the sixth floor of the general building of the National ChengChi University, Taipei, Taiwan (Figure 7). The testing area size is 33 m × 85 m.

### 3.2. Magnetic Field Fingerprint Map Results

**Figure 8:**
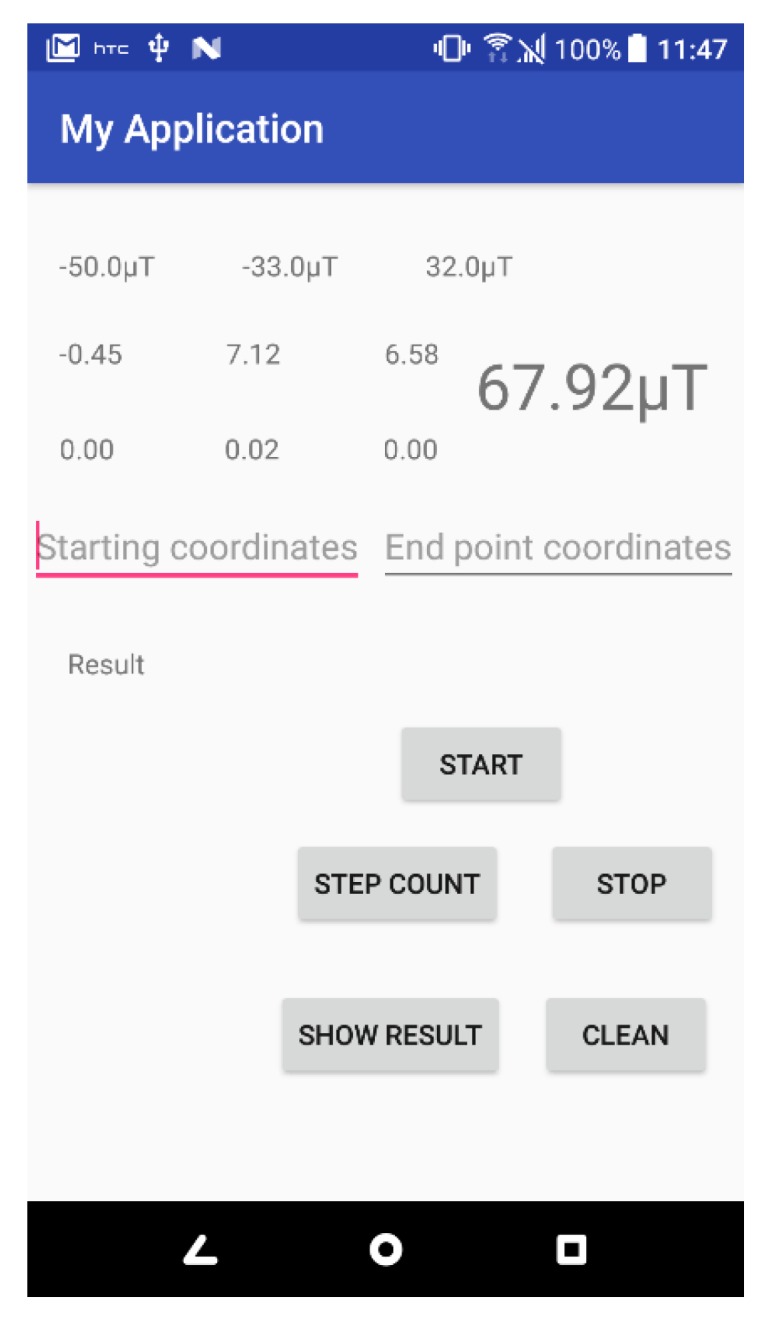
Magnetic field value recording interface.

**Figure 9:**
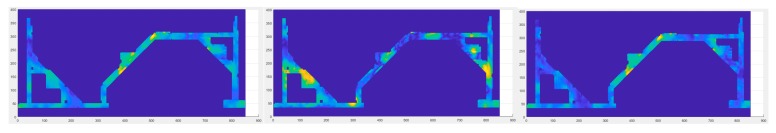
Magnetic fingerprint map of three elements in the testing area (μT).

The application of a programming interface that was developed in Android Studio is presented in Figure 8. The interface also determines the number of steps and calculates the step length. The raw data of the magnetic field fingerprint values were collected at a spacing of 40 cm apart along the corridor by a Samsung S8 smartphone in this study. It obeyed Potortì et al.’s proposed standard procedure [25]. The interpolation method was used to create three magnetic fingerprint maps, which in the gravitational direction, horizontal direction, and resultant direction of the magnetic field. The indoor magnetic field data of all corridors on the sixth floor of the general building were collected. Moreover, the interpolation method was used to create three magnetic maps of the magnetic fingerprint map (Figure 9).

## 4. Positioning Results

**Figure 10:**
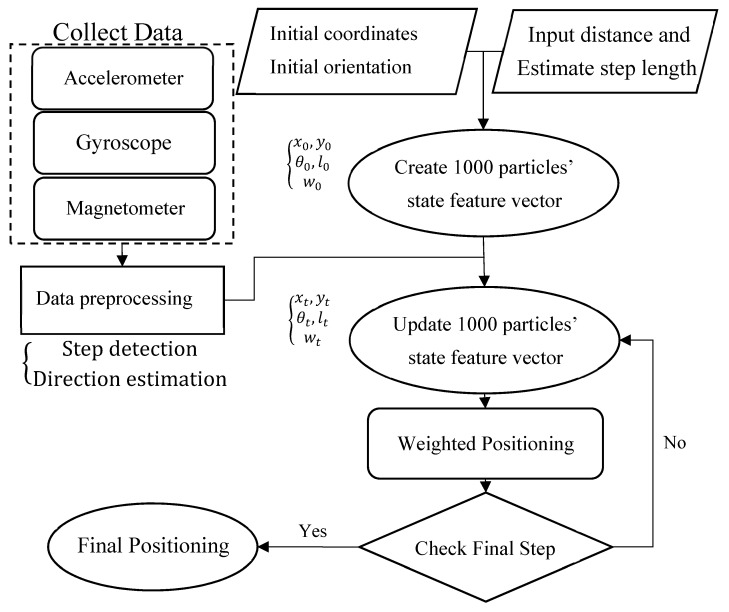
Positioning process involving the particle filter method and PDR.

In the positioning process, the particle filter method was combined with the PDR algorithm to determine the position and value of the magnetic fingerprint. Then, the result was matched with the experimental area magnetic fingerprint database to find the best position (Figure 10).

**Figure 11:**
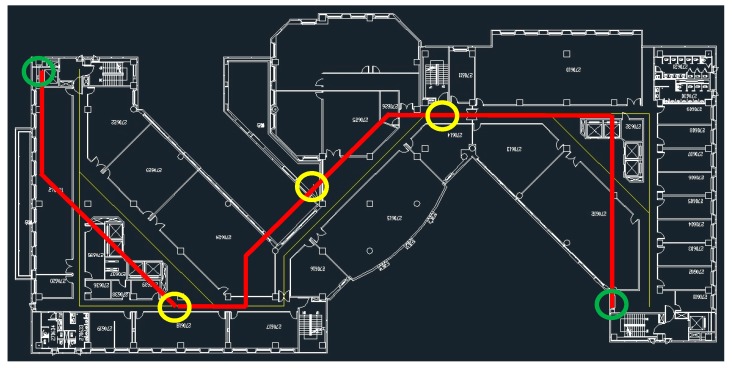
Route map of the experimental area.

In this section, we compared and analysed the results of different weighting methods of the particle filter—Xie’s method [24] and the modified weighting method proposed in this study. The testing route is displayed in Figure 11. The route has two different angles (90° and 45°) and left and right turns. A route with a width of less than 2 m is a challenging route because an accurate positioning accuracy is required to complete the entire route. In this study, some checkpoints (yellow circles) were set up on the way not only to confirm the closure difference at endpoints (green circles) but also to check if there are excessive offsets in the path.

### 4.1. Comparison of Different Methods

Ten participants followed the designed route in a sequence. Then, Xie’s method and the proposed modified particle filter method were used to compute the locations of participants. The computation results are presented in Table 3 and Table 4.

**Figure 12:**
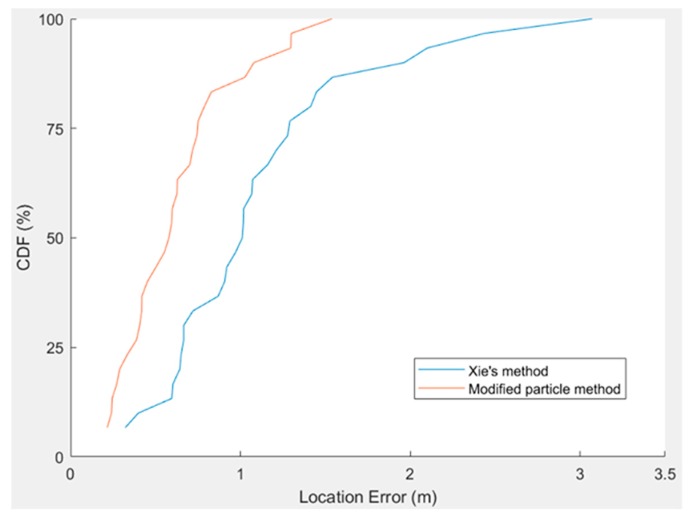
Cumulative distribution function illustration of the location error for Xie’s method and the modified particle method.

Table 2 and Table 3 reveal that the mean closure error of Xie’s method is 1.305 m and of the modified particle filter method is 0.871 m. The cumulative distribution functions of the two methods are illustrated in the same graph that is shown in Figure 12. Under a closure error of 1 m, location errors obtained using the modified particle filter method and Xie’s method are 85% and 75%, respectively.

### 4.2. Results of the Modified Particle Filter Method

**Figure 13:**
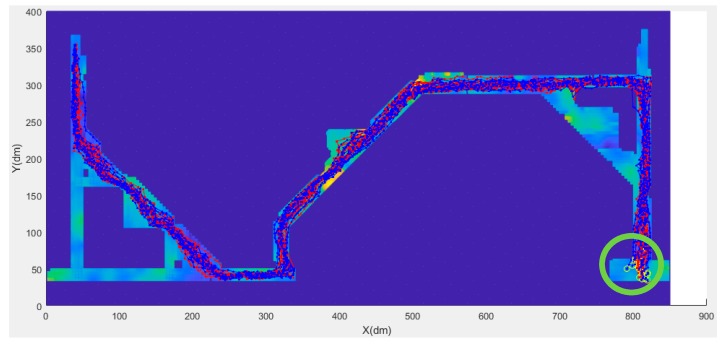
Test result of 30 users (forward).

**Figure 14:**
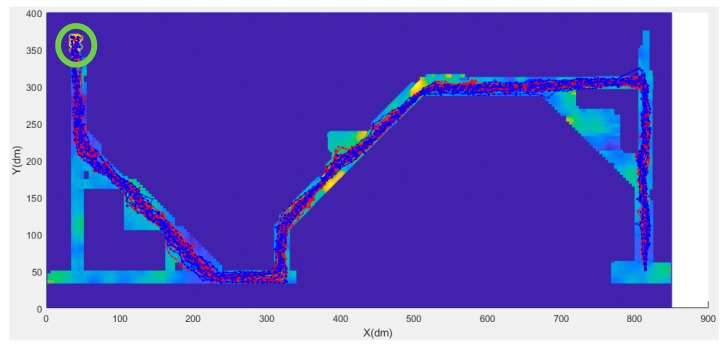
Test result of the 30 users (backward).

Based on the comparison results of Xie’s method and the modified particle filter method, an experiment was conducted in which 15 participants, including male and female participants, walked back and forth along the design route of the experimental area. All data were processed using the modified particle filter method, and results are shown in Table 5 and Figure 13 and Figure 14. The results of the male participants were poor. Moreover, the average positioning error for male participants was approximately 1 m and that for female participants was in the range of 0.6 to 0.7 m.

Figure 13 and Figure 14 present the trajectory of the 30 people in the experiment. In the figures, the blue line segment represents male, and the red line segment represents female. The green circle at the end of the positioning track indicates the location at which the last positioning ended for each experimenter’s route.

## 5. Conclusions

The results of this study reveal that the indoor positioning accuracy is in the range of 0.6 to 0.8 m when the proposed method is used. As shown in Table 6, the proposed method is better than other related studies. In this study, the modified particle filter method and indoor magnetic field map were combined to optimise the PDR algorithm. This combination not only reduces the construction cost but also improves the positioning efficiency and accuracy.

## Figures and Tables

**Table 1:** Overview of the indoor positioning technologies [1].

Technology	Typical Accuracy	Typical Coverage (m)	Typical Measuring Principle	Typical Application
Cameras	0.1 mm–dm	1–10	Angle measurements from images	Metrology, robot navigation
Infrared	cm–m	1–5	Thermal imaging, active beacons	People detection, tracking
Tactile and Polar Systems	μm–mm	3–2000	Mechanical, interferometry	Automotive, metrology
Sound	cm	2–10	Distances from time of arrival	Hospital, tracking
WLAN/Wi-Fi	m	20–50	Fingerprinting	Pedestrian navigation, lbs
RFID	dm–m	1–50	Proximity detection, fingerprinting	Pedestrian navigation
Ultra-Wideband	cm–m	1–50	Body reflection, time of arrival	Robotics, automation
High Sensitive GNSS	10 m	global	Parallel correlation, assistant GPS	Location based services
Pseudolites	cm–dm	10–1000	Carrier phase ranging	Gnss challenged pit mines
Other Radio Frequencies	m	10–1000	Fingerprinting, proximity	Person tracking
Inertial Navigation	1%	10–100	Dead reckoning	Pedestrian navigation
Magnetic Systems	mm–cm	1–20	Fingerprinting and ranging	Hospital, mines
Infrastructure Systems	cm–m	building	Fingerprinting, capacitance	Ambient assisted living

**Table 2:** Comparison more frequent use of Wireless Indoor Positioning.

Technology	Accuracy	Advantages	Disadvantages
Bluetooth/iBeacon	cm–m	Low power consumption Small equipment	Software correction required Poor stability
RFID	dm–m	Low cost Short reaction time	Low transmission Poor anti-interference ability
Wi-Fi	m	Large-scale positioning High anti-interference ability	High power consumption Low precision
Zigbee	m	Low power consumption High efficiency	Slow information transfer Low precision
UWB	cm	High precision High security	High cost High power consumption

**Table 3:** Xie’s method.

No.	Closure Error (m)	Relative Precision	Check Point 1 Error (m)	Check Point 2 Error (m)	Check Point 3 Error (m)
1	0.346	1/402	1.067	0.666	0.651
2	0.958	1/145	0.869	1.211	0.399
3	2.145	1/65	0.971	1.017	0.596
4	2.628	1/53	1.011	1.542	0.322
5	0.487	1/286	0.721	2.435	3.071
6	1.126	1/123	0.603	1.019	0.643
7	1.210	1/115	0.283	1.448	0.666
8	1.293	1/107	1.072	1.414	0.920
9	1.971	1/71	1.964	1.291	2.100
10	0.889	1/156	1.160	0.907	1.278
Mean	1.305	1/106	0.972	1.295	1.065

**Table 4:** Modified particle filter method.

No.	Closure Error (m)	Relative Precision	Check Point 1 Error (m)	Check Point 2 Error (m)	Check Point 3 Error (m)
1	0.545	1/255	1.297	1.299	0.599
2	0.585	1/238	0.407	0.388	0.829
3	2.174	1/64	0.272	0.245	0.334
4	0.990	1/140	0.552	0.744	0.216
5	0.303	1/458	0.241	0.419	0.629
6	1.095	1/127	0.702	0.718	0.788
7	0.266	1/523	0.202	1.079	0.626
8	0.884	1/157	0.452	1.539	0.503
9	0.424	1/328	0.289	1.027	0.751
10	1.448	1/96	0.595	0.579	0.420
Mean	0.871	1/160	0.501	0.804	0.569

**Table 5:** Results of 15 participants for each male and female.

	Male (m)	Female (m)
Average closure error (Go)	1.101	0.753
Average closure error (Back)	0.580	0.318
Average error in the path	0.643	0.663
Relative accuracy in the path	1/216	1/210

**Table 6:** Comparison between the indoor positioning accuracy of different methods.

Literature Authors	Positioning Method	Accuracy (m)	Precision (m)	Testing Area Size
Le Grand and Thrun, 2012 [23]	Particle Filter	0.95	0.7 for line 1.2 for circle	7 m × 7 m 4 m × 4 m
Xie et al., 2015 [24]	Particle Filter	1.0	80% within 1.6 50% within 0.8	72 m × 64 m
Lee, Ahn, and Han, 2018 [26]	Deep Leaning-based Classification	1.7	80% within 2 50% within 0.8	15 m × 22 m 15 m × 65 m
Huang et al., 2018 [5]	Particle Filter	1.13	80% within 1.5 50% within 1	1.5 m × 10 m
This Study	Modified Particle Filter	0.7	80% within 1 50% within 0.64	33 m × 85 m
